# Effects of SPA4 peptide on lipopolysaccharide‐disrupted lung epithelial barrier, injury, and function in a human cell system and mouse model of lung injury

**DOI:** 10.14814/phy2.15353

**Published:** 2022-07-15

**Authors:** Asif Alam Chowdhury, Nachiket M. Godbole, Neha Chataut, Stanley Kosanke, Karla Rodgers, Shanjana Awasthi

**Affiliations:** ^1^ Department of Pharmaceutical Sciences University of Oklahoma Health Sciences Center Oklahoma City OK USA; ^2^ Division of Comparative Medicine University of Oklahoma Health Sciences Center Oklahoma City OK USA; ^3^ Department of Biochemistry and Molecular Biology University of Oklahoma Health Sciences Center Oklahoma City OK USA

**Keywords:** epithelial barrier, lipopolysaccharide, lung injury

## Abstract

Disrupted epithelial barrier, fluid accumulation, inflammation, and compromised physiology are hallmarks of lung injury. Here we investigated the structural stability of the Toll‐like receptor‐4 (TLR4)‐interacting SPA4 peptide, its effect on *Pseudomonas aeruginosa* lipopolysaccharide (LPS)‐disrupted epithelial barrier in a human cell system, and lung injury markers in a mouse model of LPS‐induced lung inflammation. The structural properties of SPA4 peptide were investigated using circular dichroism and UV–VIS spectroscopy. The transepithelial electrical resistance (TEER), an indicator of barrier function, was measured after the cells were challenged with 1 μg/ml LPS and treated with 10 or 100 μM SPA4 peptide. The expression and localization of tight junction proteins were studied by immunoblotting and immunocytochemistry, respectively. Mice were intratracheally challenged with 5 μg LPS per g body weight and treated with 50 μg SPA4 peptide. The lung wet/dry weight ratios or edema, surfactant protein‐D (SP‐D) levels in serum, lung function, tissue injury, body weights, and temperature, and survival were determined as study parameters. The spectroscopy results demonstrated that the structure was maintained among different batches of SPA4 peptide throughout the study. Treatment with 100 μM SPA4 peptide restored the LPS‐disrupted epithelial barrier, which correlated with the localization pattern of Zonula Occludens (ZO)‐1 and occludin proteins. Correspondingly, SPA4 peptide treatment helped suppress the lung edema and levels of serum SP‐D, improved some of the lung function parameters, and reduced the mortality risk against LPS challenge. Our results suggest that the anti‐inflammatory activity of the SPA4 peptide facilitates the resolution of lung pathology.

## Graphical abstract

The TLR4‐interacting SPA4 peptide restores the lipopolysaccharide‐disrupted alveolar epithelial barrier function and distribution of tight junction proteins. Therapeutic treatment with SPA4 peptide reduces lung edema and inflammation, and improves lung function parameters and survival in a mouse model of lipopolysaccharide‐induced lung inflammation.

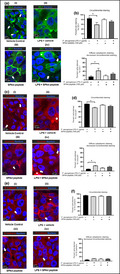

## 1 BACKGROUND

Acute respiratory distress syndrome (ARDS) is a significant public health problem worldwide, and is associated with increased morbidity and mortality (Fanelli et al., 2013). Environmental factors, toxic agents, and pathogens and pathogen‐derived ligands can induce acute lung inflammation, pulmonary edema, and hypoxia, which are common hallmarks of lung injury in ARDS. The ARDS condition can lead to failure of the pulmonary and cardiovascular systems and other organs. Patients with ARDS are admitted to intensive care units and are given oxygen therapy, nutrition support, and antibiotics. There is no specific therapeutic strategy available for the treatment of ARDS.

Pulmonary surfactant, a mixture of lipids and proteins, is synthesized and secreted by type II lung alveolar epithelial cells, and is responsible for the maintenance of lung function (Wu et al., 2003). Among four surfactant proteins, surfactant protein‐A (SP‐A) and SP‐D are critical for pulmonary host defense. Total protein contributes to 10% of total surfactant, and SP‐A is the major surfactant protein (Pastva et al., 2007). Patients with ARDS have low levels of secreted SP‐A in their alveoli (Kuroki et al., 1998). While screening the functional relevance of Toll‐like receptor‐4 (TLR4)‐interacting regions of SP‐A, the SPA4 peptide (amino acid sequence: GDFRYSDGTPVNYTNWYRGE) was identified in our laboratory (Awasthi et al., 2011). The SPA4 peptide interacts with LPS‐activated TLR4 and attenuates TLR4‐induced inflammation in cell systems and in mouse models of lung inflammation induced by intraperitoneal and intratracheal challenge with *Escherichia coli* O111:B4 LPS (Awasthi et al., 2015; Rakhesh et al., 2012; Ramani et al., 2013; Ramani & Awasthi, 2015). In the present work, we used *Pseudomonas aeruginosa* 10 LPS to delineate the effect of SPA4 peptide against LPS from another Gram‐negative bacteria. Although structural differences have been noted in LPS derived from *E. coli* and *P. aeruginosa*, the TLR4‐immunobiology is not well understood (Ernst et al., 2003, 2007; Lam et al., 2011). An inflammatory response against infectious and inflammatory stimuli leads to the release of endogenous stress ligands or damage‐associated molecular patterns (DAMPs). Some of these DAMPs, such as heat shock protein, high mobility group box‐1, fibrinogen, fibronectin, hyaluronan, and heparan sulfate, can also activate TLR4 (Erridge, 2010).

The objective of the present study was to evaluate the structural stability of SPA4 peptide and its activity in a human cell system of LPS‐disrupted epithelial barrier function and in a mouse model of LPS‐induced lung inflammation. The transepithelial electrical resistance (TEER) and expression and localization of tight junction proteins were monitored in a human lung alveolar epithelial cell system. We also investigated the lung injury parameters (edema, levels of SP‐D in serum, and inflammation), physiology (lung function, body temperature, and body weights), and survival of LPS‐challenged, SPA4 peptide‐treated mice.

Our results demonstrated that the structure of SPA4 peptide remains stable over time among different batches. One of the characteristics of ARDS is dysregulation of the epithelial barrier, which contributes to the accumulation of inflammatory mediators and pulmonary edema (Fanelli et al., 2013; Wheeler & Bernard, 2007). The TEER (Ohm·cm^2^), an indicator of epithelial barrier, was reduced in human lung alveolar epithelial cells against LPS stimuli. Therapeutic treatment with the SPA4 peptide restored the LPS‐disrupted epithelial membrane barrier in a dose‐dependent manner, as demonstrated by an improvement in TEER (Ohm·cm^2^) values and localization of tight junction proteins. Correspondingly, intratracheal administration of SPA4 peptide restored the lung wet/dry weight ratio or edema, reduced the levels of SP‐D in serum and inflammation and injury in lung tissues, and improved functional parameters in LPS‐challenged mice. The SPA4 peptide‐treated mice were also at low risk of mortality. Together, these results showed that therapeutically administered SPA4 peptide helps restore lung homeostasis and function, and alleviates LPS‐induced lung injury.

## 2 METHODS

### 2.1 SPA4 peptide

The SPA4 peptide (amino acid sequence: GDFRYSDGTPVNYTNWYRGE) was synthesized at Genscript. Mass spectroscopy and high‐performance liquid chromatography confirmed the molecular weight and purity of the peptide, respectively. The SPA4 peptide preparations were ≥95% pure. The stock solutions of separate batches of SPA4 peptide were prepared at 834.2 μM concentration in endotoxin‐free water and stored at −20°C. Aliquots of stock solutions of SPA4 peptide were freshly thawed at the time of assessment of structural characteristics of SPA4 peptide. The SPA4 peptide aliquots were thawed not more than three times for experiments performed in a cell culture system or in a mouse model of LPS‐induced lung inflammation.

### 2.2 Cell culture system

Human lung alveolar epithelial cells (H441; ATCC) were cultured in McCoy's 5A culture medium containing 5% fetal bovine serum and 50 μg/ml gentamicin (Thermo Fisher Scientific) at 37°C in a 5% CO_2_ incubator.

### 2.3 Animals

Five‐to‐six‐week‐old female C57BL6 mice were purchased from Jackson Laboratory. Mice were acclimated for at least 1 week before their inclusion in an experiment. Mice were randomly assigned to a study group in separate experiments performed on different occasions. Up to five mice, assigned to a particular group, were housed together. Mice were given food and water ad libitum throughout the study. The animal studies were approved by the Institutional Animal Care and Use Committee (Protocol numbers: 17‐081‐CHR and 20‐054‐SCHIR) at the University of Oklahoma Health Sciences Center (OUHSC).

### 2.4 Sterility and purity of SPA4 peptide

Sterility of stock solutions was assessed by plating 50 μg of SPA4 peptide solution on trypticase soy agar plates. 50 μg of the peptide was equivalent to the administered dose in mice. Inoculated agar plates were incubated at 37°C overnight. The next day, the plates were observed for the appearance of any bacterial growth.

To assess any detectable amount of endotoxin, the SPA4 peptide solutions were subjected to Limulus amebocyte lysate (LAL) testing using a commercially available kit (Endosafe; Charles River Laboratories). The Endosafe cartridge can detect endotoxin levels from 0.05–5.0 EU/ml. It uses a kinetic chromogenic assay to measure color intensity, which is proportional to endotoxin concentration. An aliquot of 25 μl of SPA4 peptide solution was taken in the sample reservoir of the cartridge. The endotoxin levels were determined by the resulting kinetic values after analyzing against an internally archived standard curve according to the manufacturer's guidelines. One endotoxin unit (EU) detected is equivalent to 0.111 ng of *E. coli* (O113:H10:K negative)‐derived LPS. The endotoxin concentration was determined in terms of ng/ml and ng/μg amounts of SPA4 peptide.

### 2.5 Circular dichroism (CD) spectroscopy

The SPA4 peptide solution (83.4 μM) was prepared by diluting the stock solution with 75% methanol in endotoxin‐free water. Spectra were recorded on a Jasco J‐715 CD spectropolarimeter with a PTC‐348 WI Peltier temperature controller (Jasco Corp) as described earlier (Awasthi et al., 2015; Godderz et al., 2003; Sreerama & Woody, 2000).

### 2.6 UV–VIS spectroscopy

The UV–VIS spectroscopy was performed with three separate batches of SPA4 peptide stock solutions. Briefly, 2 μl of SPA4 peptide solution was loaded per well of a micro‐volume glass slide plate (0.5 mm vertical optical path; Take 3 plate, Biotek) in triplicate. Absorbance was read at the interval of every 5 nm within a range of 200–350 nm wavelength (Biotek). An equivalent volume of water in triplicate wells served as a negative control. The data were collected using Gen 5 software (Biotek).

### 2.7 Measurement of transepithelial electrical resistance (TEER) in a human lung alveolar epithelial cell system

#### 2.7.1 Membrane barrier formation

About 1 × 10^5^ cells were seeded on the apical side of the Transwell filter insert (pore size 1.0 μm, surface area 0.33 cm^2^; Millipore Sigma). An insert with the cell culture medium alone was included as a blank control. The apical and basolateral chambers of the insert contained 100 and 600 μl culture medium, respectively. The EVOM2 Epithelial Voltohmmeter (World Precision Instrument) with STX2 electrode was used to measure TEER. Cell culture medium was changed at the same time in both chambers after every 24 h. The following formula was used for calculating the TEER (Ohm·cm^2^):

- TEER (Ohm) = TEER in wells with cells (Ohm) – TEER value in blank well without cells (Ohm)
- TEER (Ohm·cm^2^) = TEER (Ohm) calculated in step A × area of insert (cm^2^)

Two consecutive maximum values of 70–80 Ohm·cm^2^ were considered for uniform membrane barrier formation by H441 cells.

#### 2.7.2 SPA4 peptide activity on LPS‐induced membrane barrier disruption

After the barrier formation was confirmed, the cells were challenged with 1 μg/ml *P. aeruginosa* 10‐derived LPS (purified by gel filtration chromatography, protein content ≤3%; Millipore Sigma) in apical and basolateral chambers of the inserts. The cells were treated with 10 or 100 μM SPA4 peptide post 1 h LPS‐challenge. Vehicle‐treated cells were included as a control. The TEER measurements were recorded in triplicate at 0, 6, 24, and 48 h of LPS challenge, and calculated in terms of Ohm·cm^2^ as described above. All calculated TEER values (Ohm·cm^2^) at different time points were normalized with 0‐h readings for each well.

#### 2.7.3 Localization of tight junction (Zonula Occludens‐1 or ZO‐1, occludin), and adherens junction (E‐Cadherin) proteins

The H441 lung alveolar epithelial cells were seeded in 8‐well chambered slides at the density of 5 × 10^4^ cells per well. After 3 days, the cells were challenged with *P. aeruginosa* LPS (1 μg/ml) and treated with 100 μM SPA4 peptide 1 h post LPS challenge. After 24 h of LPS challenge, the cells were fixed with 4% paraformaldehyde (Thermo Fisher Scientific), washed, and permeabilized with 0.1% Triton‐X 100 (Acros Organics) for 10 min. Nonspecific sites were blocked for an hour by incubating the cells with 1% bovine serum albumin (BSA). The cells were then stained with Alexa Fluor 488‐conjugated ZO‐1‐specific antibody and Alexa Fluor 594‐conjugated antibody specific to occludin, (each diluted 1:100, both antibodies from Thermo Fisher Scientific) for 90 min, and 1:50 diluted Alexa Fluor 594‐conjugated antibody specific to E‐Cadherin (Cell Signaling Technology) for 3 h. After washing with phosphate‐buffered saline, the cells were stained with 1:2000 diluted Hoechst 33342 dye (Thermo Fisher Scientific). The immunostained cells were then mounted using ProLong Gold anti‐fade reagent. The slides were then sealed and stored at 4°C before microscopic analysis. Unstained cells served as a control. The localization pattern was analyzed in at least 100 cells of each group.

### 2.8 Expression of ZO‐1, occludin, and E‐Cadherin proteins

The H441 cells were seeded in 24‐well tissue culture plates at the density of 3 × 10^5^ cells per well, challenged with 1 μg/ml *P. aeruginosa* LPS at 0 h, and treated with 100 μM SPA4 peptide or an equivalent volume of the vehicle at 1 h. After 24 h of incubation, the cells were lysed in RIPA lysis buffer (Rockland Immunochemicals) containing 0.5 μg/ml leupeptin, 0.68 μg/ml pepstatin, 0.2 mM phenylmethylsulfonyl fluoride, 0.01 mM sodium fluoride, and 0.2 mM sodium orthovanadate. Cytosolic and membrane fractionation was performed using a membrane protein extraction kit (Biovision). The total protein concentration was measured in diluted cell lysates and cytosolic and membrane fractions by bicinchoninic acid assay (Thermo Fisher Scientific).

Total protein of whole cell lysate (1 μg), cytosolic (0.2–1 μg), and membrane (0.5–1 μg) fractions was resolved on 4%–20% Tris‐glycine sodium dodecyl sulfate‐polyacrylamide gel electrophoresis (SDS‐PAGE) gels. Separated proteins were then transferred onto nitrocellulose membranes. Membranes with transferred proteins were incubated with 5% solution of nonfat powdered milk in tris‐buffered saline (Blocker BLOTTO, Thermo Fisher Scientific) for blocking the non‐specific sites, followed by incubation with primary antibodies specific for ZO‐1, occludin, and E‐Cadherin, each diluted 1:1000 in tris‐buffered saline, for 2–18 h. The membranes were washed and incubated with horseradish peroxidase‐conjugated anti‐rabbit IgG antibody (diluted 1:1000). The immunoreactive bands were visualized using SuperSignal West Pico Plus or Femto substrate solutions (Thermo Fisher Scientific) on a Chemidoc Imaging system (Bio‐Rad Laboratories). The membrane was then stripped at 50°C for 30 min in stripping buffer (10 ml of 10% SDS, 6.25 ml of 0.5 M Tris, and 350 μl of β‐mercaptoethanol), washed, and again probed with 1:1000 diluted anti β actin antibody followed by 1:1000 diluted horseradish peroxidase‐conjugated secondary antibody. The densitometric analysis of immunoreactive bands of tight junction and adherens junction proteins and β actin was performed using ImageJ software (NIH). The arbitrary densitometric units of respective proteins were normalized with those of β actin.

### 2.9 Assessment of SPA4 peptide activity in a mouse model of *P. aeruginosa* LPS‐induced lung inflammation and injury

Mice were intratracheally instilled with *P. aeruginosa* 10‐derived LPS (5 μg/g body weight) and treated with 50 μg SPA4 peptide or an equivalent volume of the vehicle at 1 h post LPS‐challenge via intratracheal route, as described earlier (Awasthi et al., 2020). Mice were euthanized at different times, and blood and whole lung or lung tissue specimens were collected. Whole lung was immediately fixed in 10% formalin for histopathology or was used for an assessment of lung wet/dry weight ratios. Lung tissues and blood collected for analysis of other parameters were processed as described below.

#### 2.9.1 Lung wet/dry weight ratios

The weights of wet harvested lung were measured immediately. Lungs were then dried at 60°C for 48 h in a vacuum oven (Fisher Scientific) and weighed to obtain dry lung weight. Lung wet‐to‐dry weight ratios (lung edema) were calculated and compared among different groups of mice.

#### 2.9.2 Lung function analysis

In separate sets of experiments, mice were anesthetized with ketamine and xylazine intraperitoneally after 4 and 24 h of LPS challenge. Mouse tracheas were exposed and cannulated with an 18‐gauge blunt needle. Mice were then given pancuronium bromide (~0.1 μg per g body weight; Sigma) via the intraperitoneal route to prevent spontaneous breathing. Mice were immediately connected to the ventilator via Y‐tubing of Flexivent (SciReq). Measurements of lung function were obtained using a predefined script. The tasks used were Deep Inflation (27 cm H_2_O, 6 s), Snapshot‐150 (10 ml/Kg, 1.25 s), Quick Prime‐3 (3 ml/kg, 3 s), and Pressure‐Volume loops (27 cm H_2_O, 15.92 s). The script was run for four‐to‐five times on each mouse. The data output from these tasks included estimate of inspiratory capacity (ml, A), area (ml·cm H_2_O), coefficient of determination (COD), COD for the constant phase model (CODcp), COD for the Salazar‐Knowles model (CODsk), compliance (ml/cm H_2_O, Crs), quasi‐static compliance (Cst, ml/cm H_2_O), elastance (cm H_2_O/ml, Ers), tissue dampening (cm H_2_O/ml, G), tissue elasticity (cm H_2_O/ml, H), inspiratory capacity (ml, IC), curvature of the upper portion of the deflation limb of the pressure‐volume curve (K), airway resistance (cm H_2_O.s/ml, Rn), and total respiratory system resistance (cm H_2_O.s/ml, Rrs). Measurements of each of the first and last cycles were discarded to remove any transients in the calculations, per published report (Mora et al., 2000).

#### 2.9.3 Survival

Mice were followed for survival for 10 days. Mice were anesthetized using isoflurane anesthesia; back skin temperatures and body weights were then recorded at regular intervals. Skin temperature was measured on the backs of the mice using a calibrated 153 IRB infrared thermometer (BIOSEB).

#### 2.9.4 Collection of serum

The blood was collected from anesthetized mice by cardiac puncture, and was centrifuged at 472 x *g* for 15 min at 4°C. The serum specimens were stored at −80°C until further analysis.

#### 2.9.5 Measurement of SP‐D in serum

The levels of SP‐D were determined in serum as a measure of intravascular leakage (Gaunsbaek et al., 2013). Diluted serum specimens were subjected to an enzyme linked immunosorbent assay (ELISA; R&D Systems) for measurement of SP‐D levels. Briefly, microwells were coated with 4 μg/ml capture antibody overnight at 4°C. After washing the wells, the nonspecific sites were blocked with 1% BSA solution in Dulbecco's phosphate‐buffered saline (DPBS) for 1 h at room temperature. The wells were washed and incubated with the mouse SP‐D standard (62.5–4000 pg/ml) and serum specimens (1:10 and 1:50) diluted in 1% BSA solution in DPBS, overnight at 4°C. The next day, the wells were washed and incubated with 125 ng/ml detection antibody. The immune complex was then incubated with 1:40 diluted streptavidin‐horse radish peroxidase for 2 h at room temperature, and developed by adding tetramethylbenzidine substrate solution (Sigma). The reaction was stopped by adding 2 N H_2_SO_4_. Finally, the optical density was read spectrophotometrically at 450 and 540 nm, and subtracted per the manufacturer's instructions to correct for optical imperfections in the plate.

### 2.10 Histopathology

The lungs were harvested after 4 and 24 h of LPS challenge and fixed in 10% formalin. Whole lungs were also collected from all surviving mice at the end of the survival study, and formalin‐fixed. After 24 h, the fixed lungs were transferred into 75% ethanol. The lungs were then dehydrated, infiltrated, embedded into paraffin, sectioned, and stained with hematoxylin and eosin (H & E) at the Stephenson Cancer Center Tissue Pathology Core Laboratory, OUHSC, Oklahoma City, OK. The H & E‐stained lung tissue sections were then examined by a board‐certified veterinary pathologist. The intravascular/intra‐alveolar influx of inflammatory cells, consisting mostly of neutrophils and a lesser number of macrophages, was scored as mild (a small number of mostly intravascular neutrophils), moderate (a mixture of both intravascular and intra‐alveolar neutrophils), or marked (a mixture of intravascular and intra‐alveolar neutrophils with evidence of consolidation due to a marked, focal, intra‐alveolar influx of inflammatory cells).

### 2.11 Statistical analysis

Data were analyzed using *t*‐test, one‐way or two‐way Analysis of Variance (ANOVA), followed by Tukey's post hoc analysis for multiple comparisons, or Fisher's exact test for comparison of proportions in two categories between the study groups (GraphPad Prism software). Survival proportions were compared using Mantel–Haenszel hazard ratio. A *p* value of <0.05 was considered statistically significant or otherwise indicated.

## 3 RESULTS

### 3.1 Structure of SPA4 peptide remains stable under set storage conditions

**FIGURE 1:**
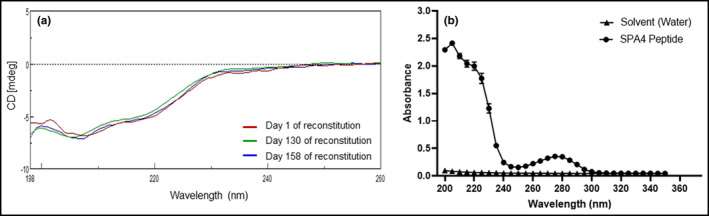
Stability of structural characteristics of SPA4 peptide. The CD spectra of SPA4 peptide diluted in 75% methanol over a period of 6 months of shelf life after reconstitution in endotoxin‐free water. The CD spectroscopy was performed at different time intervals for three separate batches of SPA4 peptide. An overlay of CD spectra from one representative batch of SPA4 peptide is shown in (a). The UV–VIS spectroscopy was performed for three separate batches of SPA4 peptide over a wavelength range of 200–350 nm. The mean (±standard error of mean, SEM) absorbance values of a single batch of SPA4 peptide obtained on days 79, 96, 153, 170, and 180 are shown in (b). Endotoxin‐free water (solvent) was included as control.

The SPA4 peptide stock solutions were sterile and remained free of endotoxin during storage over the six‐month shelf life after reconstitution. There was no bacterial growth at any time in stock solutions of the peptide. The LAL assay (sensitivity 0.00555 ng/ml or 0.05 EU/ml) results showed that SPA4 peptide had endotoxin levels of 0.012 ± 0.0048 (Mean ± SEM) ng/ml or 0.000006 ± 0.0000024 ng/μg peptide. As published earlier, the structural and physicochemical properties of SPA4 peptide are critical for its binding to TLR4 complex and activity (Awasthi et al., 2015, 2021). We employed two approaches, CD and UV–VIS spectroscopy, to assess any change in the structural attributes of SPA4 peptide over time. Our results demonstrate that the CD spectrum of SPA4 peptide remains consistent at different time points for three separate batch preparations over a period of 6 months after reconstitution in endotoxin‐free water (Figure 1a). The absorption spectra over the wavelength range of 200–350 nm showed a stable profile for SPA4 peptide over 6 months (Figure 1b). The endotoxin‐free water served as a blank. Maintenance of the structural attributes of different batches of SPA4 peptide demonstrates the integrity of SPA4 peptide for the respective analyses (Figure 1).

### 3.2 SPA4 peptide restores the LPS‐disrupted human lung alveolar epithelial cell barrier

**FIGURE 2:**
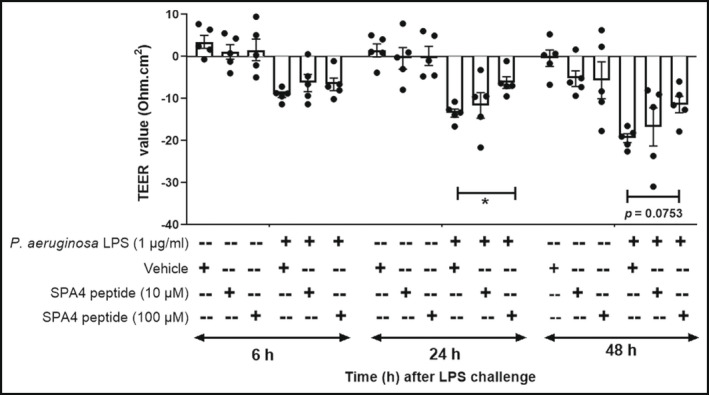
Effect of SPA4 peptide on epithelial membrane barrier function of H441 cells. The membrane barrier was formed by culturing H441 cells on filter inserts. The cells were then challenged with *Pseudomonas aeruginosa* LPS (1 μg/ml) and treated with 10 or 100 μM SPA4 peptide or the equivalent amount of vehicle 1 h post LPS challenge. The TEER measurements (Ohm·cm^2^) were obtained using an EVOM2 Epithelial Voltohmmeter at 0, 6, 24, and 48 h of LPS challenge, and were normalized with those obtained at 0 h. Normalized TEER values (Ohm·cm^2^; Mean ± SEM) from five separate experiments are shown. Statistical significance was determined by two‐way ANOVA with Tukey's post‐hoc analysis (*p < 0.05, or as indicated within the figure).

The epithelial monolayer formation was indicated by consistent TEER values and was maintained in vehicle‐treated cells. Similarly, there was no significant difference in calculated TEER values of cells treated with SPA4 peptide (10 or 100 μM) alone, over a period of 48 h. However, the *P. aeruginosa* LPS significantly reduced the TEER values (Ohm·cm^2^), indicating epithelial barrier disruption. Treatment with 10 μM SPA4 peptide only slightly improved the LPS‐disrupted epithelial cell barrier function (statistically not significant). However, treatment with 100 μM of SPA4 peptide restored the TEER values (Ohm·cm^2^) at 24 and 48 h. This restorative effect of SPA4 peptide on LPS‐disrupted epithelial barrier was pronounced after 24 h (*p* < 0.05) and 48 h (*p* = 0.0753) of LPS challenge (Figure 2).

### 3.3 SPA4 peptide restores the LPS‐induced changes in epithelial cell localization of ZO‐1 and occludin

**FIGURE 3:**
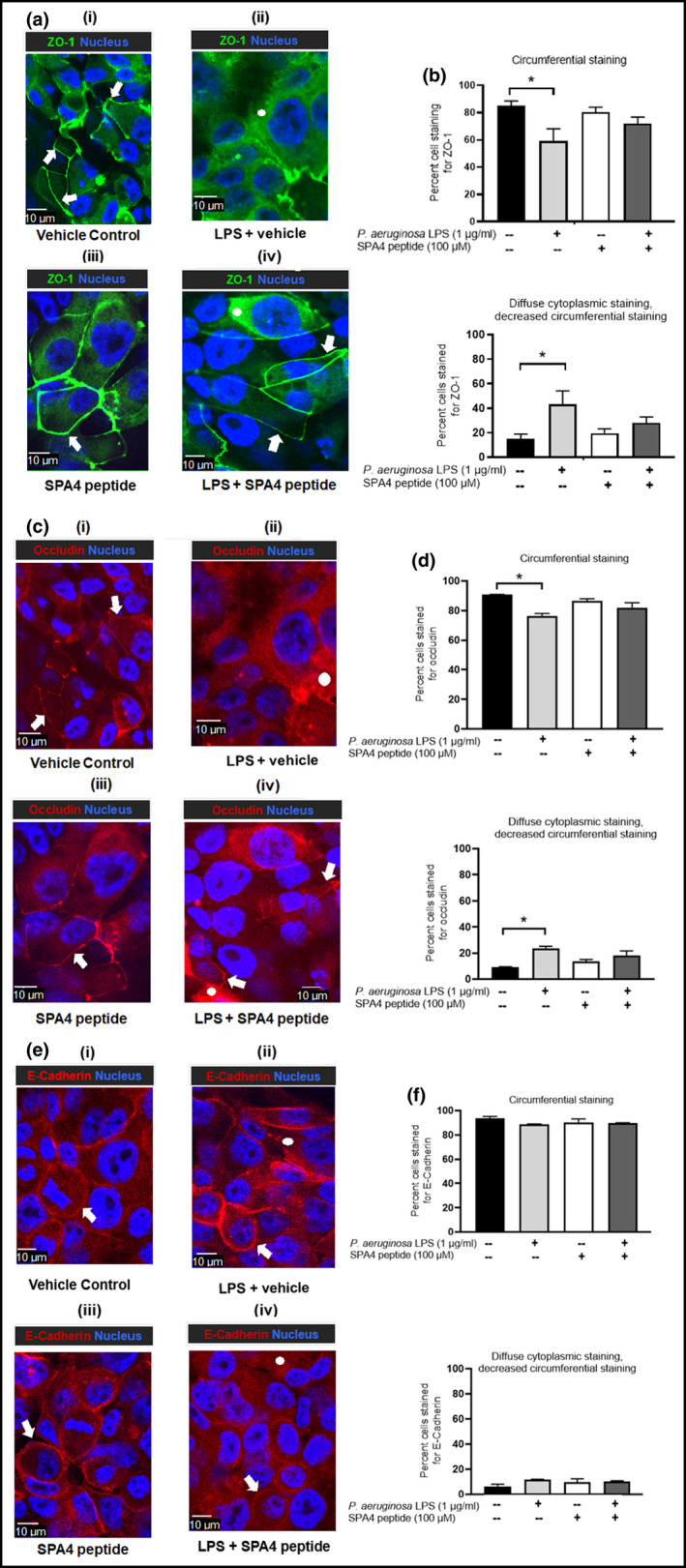
Localization of ZO‐1, occludin, and E‐Cadherin in H441 cells. The cells were challenged with 1 μg/ml *Pseudomonas aeruginosa* LPS at 0 h, treated with 100 μM SPA4 peptide or vehicle after 1 h of LPS challenge, and were immunostained for ZO‐1 (in green), occludin and E‐Cadherin (in red), and nuclear staining (in blue). Photomicrographs were collected at 60 X magnification from two separate experiments. Representative images of cells for each group are shown in (a, c, e). The staining patterns (circumferential, shown as arrows; diffused cytoplasmic staining with decreased circumferential staining shown as circles) were noted in at least 100 cells from each group. The bar charts show the Mean + SEM values of percentages of cells showing distinct staining patterns (b, d, f). Statistical significance of the data was determined using one‐way ANOVA with Tukey's post hoc analysis (*p < 0.05).

The localization of tight junction protein ZO‐1, occludin, and E‐Cadherin was studied by immunostaining and confocal microscopy. The vehicle‐treated control cells showed circumferential staining of ZO‐1 and occludin. However, a diffused cytoplasmic localization of ZO‐1 and occludin was observed with decreased circumferential staining after 24 h of LPS challenge. The SPA4 peptide treatment alone did not affect the circumferential distribution of ZO‐1 and occludin in cells. However, therapeutic treatment with SPA4 peptide stimulated circumferential localization of tight junction proteins in an increased number of LPS‐challenged cells. There was no significant effect of LPS challenge or SPA4 peptide treatment on subcellular localization pattern of E‐Cadherin (an adherens junction protein). Representative photomicrographs and the percentage of cells with circumferential and diffused cytoplasmic distribution of ZO‐1, occludin, and E‐Cadherin proteins are shown in Figure 3.

### 3.4 The expression levels of tight junction (ZO‐1, occludin) and adherens junction (E‐Cadherin) proteins are not affected after SPA4 peptide treatment

**FIGURE 4:**
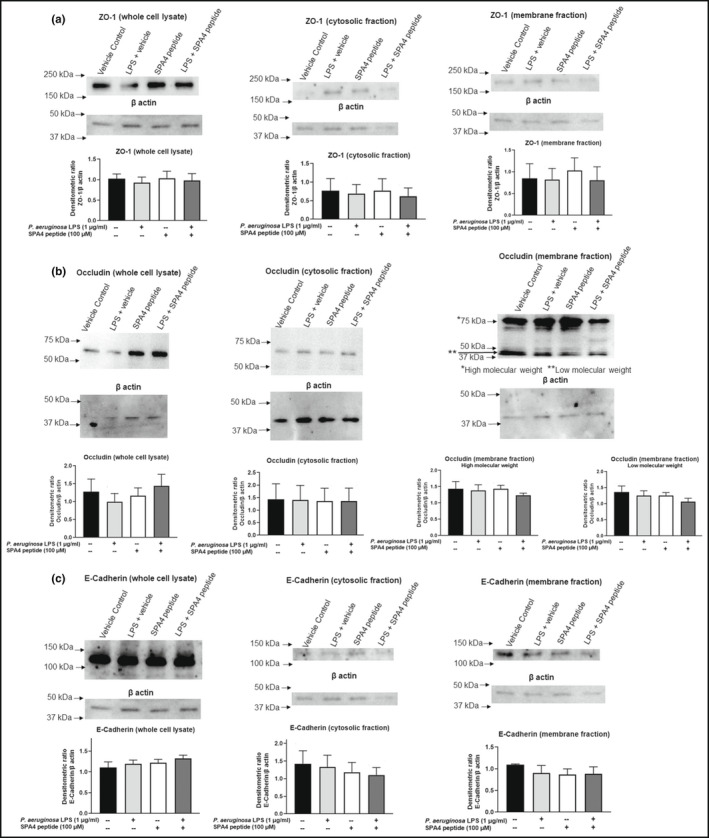
Expression of ZO‐1, occludin, and E‐Cadherin in whole cell lysates and cytosolic and membrane fraction of H441 cells. The cells were challenged with 1 μg/ml *Pseudomonas aeruginosa* LPS at 0 h, treated with 100 μM SPA4 peptide or vehicle after 1 h of LPS challenge, and harvested at 24 h. Separated proteins were immunoblotted with antibodies specific for the respective tight junction proteins and β actin (loading control). Densitometric analysis was performed on immunoreactive bands for each protein. Arbitrary densitometric units were normalized with those of β actin. The bar charts show the Mean + SEM values of densitometric ratios of each protein in separate cell fractions derived from three separate experiments performed on different occasions. Representative immunoblot images are also shown for each group.

The expression levels of the ZO‐1, occludin, and E‐Cadherin proteins were determined in whole cell lysates and cytosolic and membrane fractions of H441 cells by immunoblotting. An immunoreactive band of ZO‐1 (~220 kDa, between 150 and 250 kDa molecular weight markers), occludin (~65 kDa, between 50 and 75 kDa molecular weight markers), and E‐Cadherin (~120 kDa, between 100 and 150 kDa molecular weight markers) was detected in whole‐cell lysates and isolated cell fractions. An additional immunoreactive band (~40 kDa) was identified for occludin in the membrane fractions of H441 cells, as has been identified earlier by another independent group of researchers (Morgan et al., 2018). Only a slight decrease was observed in the expression of ZO‐1 and occludin proteins in whole‐cell lysates of LPS‐challenged cells (Figure 4a–c). When densitometric analysis results were pooled from different experiments and compared among study groups, no statistically significant changes were observed for the expression levels of ZO‐1, occludin, and E‐Cadherin proteins in whole‐cell lysates, or cytosolic/membrane fractions (Figure 4a–c).

### 3.5 Biological effects of SPA4 peptide on lung edema, pulmonary function parameters, body weight and temperature, levels of SP‐D, and survival of LPS‐challenged mice

**FIGURE 5:**
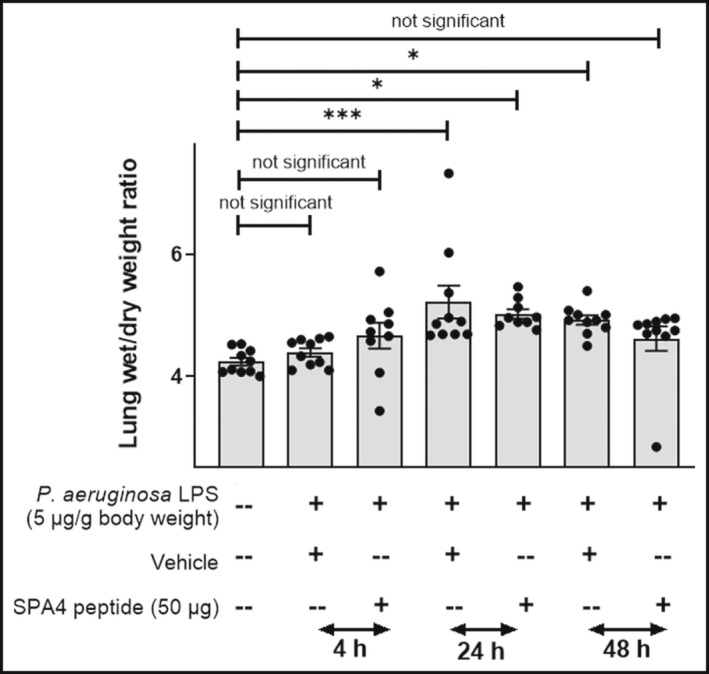
Lung wet/dry weight ratios (indicator of lung edema) in *Pseudomonas aeruginosa* LPS‐challenged and SPA4 peptide‐treated mice. Mice were intratracheally challenged with *P. aeruginosa* LPS (5 μg per g body weight) at 0 h and treated with SPA4 peptide (50 μg per mouse) or vehicle via intratracheal route at 1 h post LPS challenge. Mice were necropsied at 4, 24, or 48 h post LPS challenge. The lungs were weighed immediately and after drying at 60°C. The bars indicate mean (±SEM) lung wet/dry weight ratios. Results are derived from 9 to 10 mice per group. Statistical significance of the data was determined using one‐way ANOVA with Tukey's post hoc analysis (*p < 0.05, ***p < 0.001).

Corresponding to the disrupted epithelial barrier, increased lung wet/dry weight ratios were observed in LPS‐challenged mice. This increase in edema was more pronounced at 24 h (*p* < 0.001) and 48 h (*p* < 0.05) in lungs of LPS‐challenged, vehicle‐treated mice compared with unchallenged, untreated mice. The lung wet/dry weight ratios of mice in LPS‐challenged, SPA4 peptide‐treated group were lower, but not statistically significant, at 24 and 48 h than those in the LPS‐challenged, vehicle‐treated group, and were comparable to those in unchallenged, untreated mice at 48 h (Figure 5).

**FIGURE 6:**
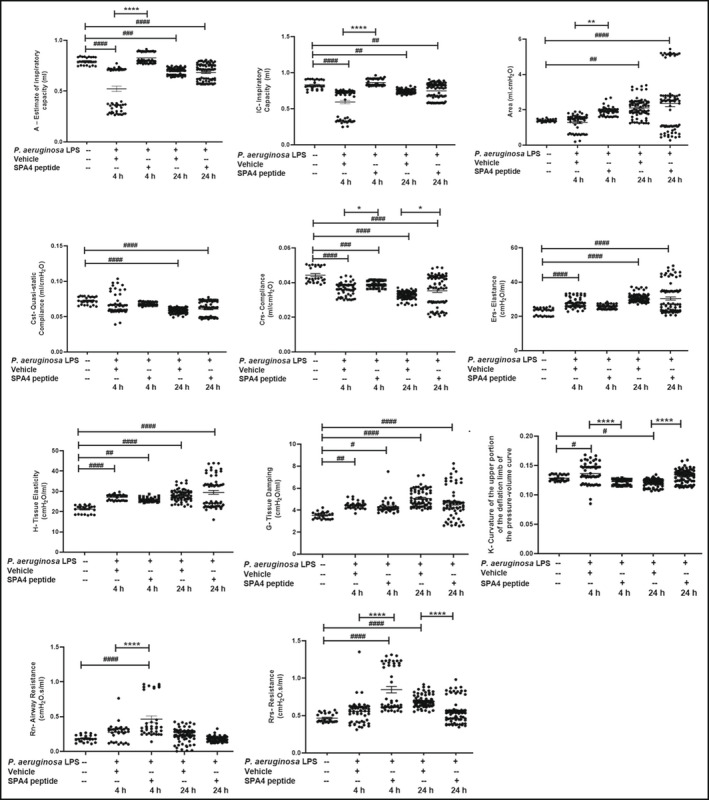
Lung function analysis of mice challenged with *Pseudomonas aeruginosa* LPS and treated with SPA4 peptide. Mice were intratracheally challenged with *P. aeruginosa* LPS (5 μg per g body weight) at 0 h and treated with SPA4 peptide (50 μg per mouse) or vehicle via intratracheal route at 1 h post LPS challenge. The lung function analysis of mice was performed at 4 and 24 h of LPS challenge. Results (mean ± SEM and individual readings) from 3–6 mice per group are shown in the figure. Statistical significance was determined among study groups using one‐way ANOVA with Tukey's post hoc analysis (^####^ *p* < 0.0001, ^###^ *p* < 0.001, ^##^ *p* < 0.005 ^#^ *p* < 0.05 vs. unchallenged, untreated mice and ****p < 0.001, **p < 0.005 and *p < 0.05 vs. LPS challenged, vehicle‐treated mice).

An assessment of pulmonary function indicated statistically significant changes in the study parameters in LPS‐challenged, vehicle‐treated mice after 4 and/or 24 h of LPS challenge compared with those in unchallenged, untreated mice. The SPA4 peptide treatment restored the LPS‐altered values of IC, A, K, Crs, and area within 4 h of LPS‐challenge (statistically different from those in the LPS‐challenged, vehicle‐treated group). The Crs and K values remained statistically different in LPS‐challenged, SPA4 peptide‐treated mice compared with those in LPS‐challenged, vehicle‐treated mice after 24 h. While the Rn and Rrs values were increased at 4 h, the Rrs values were significantly decreased in the SPA4 peptide‐treated group versus the vehicle‐treated group after 24 h (Figure 6).

**FIGURE 7:**
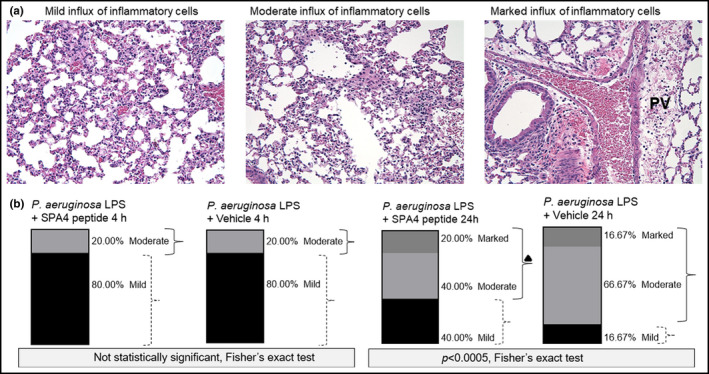
Assessment of lung inflammation in mice challenged with *Pseudomonas aeruginosa* LPS and treated with SPA4 peptide. Mice were intratracheally challenged with *P. aeruginosa* LPS (5 μg per g body weight) at 0 h and treated with SPA4 peptide (50 μg per mouse) or vehicle via intratracheal route at 1 h post LPS challenge. Whole lungs were harvested at 4 and 24 h of LPS challenge, formalin‐fixed, processed, sectioned, and stained with H & E. The H & E‐stained lung sections were studied and scored for the level of inflammation: Mild (acute inflammation characterized primarily by a mild influx of mature neutrophils within the alveolar septal capillaries and only mild evidence of edema), moderate (acute inflammation characterized primarily by a moderate influx of mature neutrophils within the alveolar septal capillaries and only mild evidence of edema), or marked (similar to moderate with the addition of an influx of mature neutrophils and edema within the peribronchiolovascular connective tissue [denoted as PV]). ▲ One animal showed moderate as well as marked levels of inflammation. Representative images are shown in (a). Distribution of lung inflammation scores in 5–6 mice per group is shown in (b).

The lung tissues from 80% of the animals in both the LPS‐challenged, vehicle‐treated, and the LPS‐challenged, SPA4 peptide‐treated study groups demonstrated only a mild level of inflammatory cell influx after 4 h of LPS‐challenge. However, moderate‐to‐marked inflammatory cell influx was noted in lungs of 83.34% (16.67% mild, 66.67% moderate, and 16.67% marked) of the LPS‐challenged, vehicle‐treated mice compared with those in 60% (40% mild, 40% moderate, and 20% marked) of LPS‐challenged, SPA4 peptide‐treated mice after 24 h of LPS‐challenge (*p* < 0.0005, Figure 7).

**FIGURE 8:**
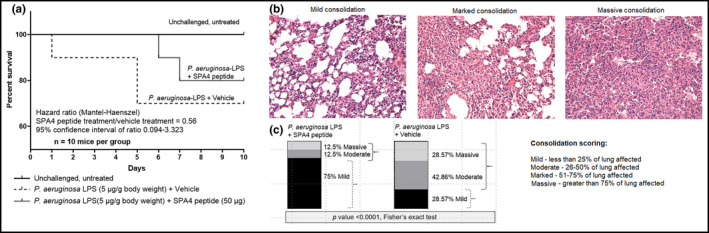
Survival of mice challenged with *Pseudomonas aeruginosa* LPS and treated with SPA4 peptide (a) and lung tissue damage (b) in mice after the 10‐day survival study. Mice were intratracheally challenged with *P. aeruginosa* LPS (5 μg per g body weight) at 0 h and treated with SPA4 peptide (50 μg per mouse) or vehicle via intratracheal route at 1 h post LPS challenge. The survival monitored in 10 mice per study group over the 10‐day period. Hazard ratio (Mantel–Haenszel) was calculated to assess any difference between the two study groups (LPS‐challenged, SPA4 peptide‐treated vs. LPS‐challenged, vehicle‐treated mice) (a). At the end of the 10‐day survival study, all surviving mice were necropsied and whole lungs were harvested, formalin‐fixed, processed, sectioned, and stained with H & E. The H & E‐stained lung sections were examined and scored for the levels of lung affected: Mild (+1, <25% of lung affected), moderate (+2, 26%–50% of lung affected), marked (+3, 51%–75% of lung affected), and massive (+4, >75% of lung affected). Representative images are shown in (b). Distribution of lung consolidation scores is shown in (c).

Survival of mice over 10 days coincided with the pulmonary function results. The LPS‐challenged mice succumbed to death between days 1 and 5. The mortality was delayed, and started only on days 6–7 in mice with a single 50‐μg dose of SPA4 peptide treatment after 1 h of LPS‐challenge. The hazard ratio of the survival curves indicates that the SPA4 peptide treatment improves the survival compared with the vehicle treatment (Mantel–Haenszel Hazard ratio 0.56 in the SPA4 peptide vs. vehicle treatment groups, 95% confidence interval of ratio 0.09404–3.323; Figure 8a).

The lungs of surviving mice were processed and stained with H & E at the end of the survival study. Histological evidence of lung inflammation and injury were noted. The terminal airways/alveolar sacs were often filled with a mixed inflammatory cell infiltrate consisting mostly of macrophages, with a lesser number of lymphocytes and neutrophils. This often resulted in mild to massive, multifocal to locally extensive, pulmonary consolidation. The bronchioles appeared to be only minimally affected. The levels of cellular infiltrates with consolidation were graded as mild (less than 25% of lung affected), moderate (26–50% of lung affected), marked (51–75% of lung affected), and massive (greater than 75% of lung affected). Seventy‐five percent of surviving mice in the LPS‐challenged, SPA4 peptide‐treated group revealed only a mild level of cellular infiltration/consolidation compared with 28.57% in the LPS‐challenged, vehicle‐treated mice (*p* < 0.0001, Figure 8b, c).

**FIGURE 9:**
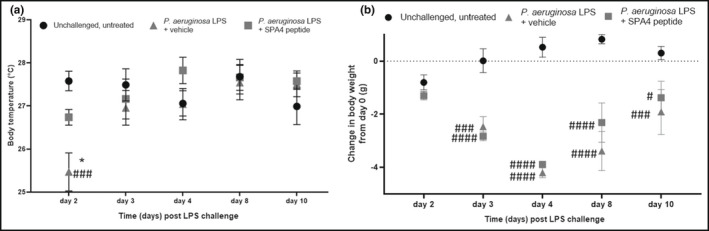
Body temperature and weight of mice challenged with *Pseudomonas aeruginosa* LPS and treated with SPA4 peptide. The body temperature was measured on the backs of the mice during the 10‐day survival study (a). The body weights were normalized with those measured prior to performing the experiment (b). Results (mean ± SEM) are from 10 mice per group included in two separate experiments. The data in (a and b) were analyzed using two‐way ANOVA with Tukey's post hoc analysis (*p < 0.05 vs. LPS‐challenged, vehicle‐treated mice, and ^####^ *p* < 0.0001, ^###^ *p* < 0.0005, ^#^ *p* < 0.05 vs. unchallenged, untreated mice).

Body temperature and body weights were monitored in surviving mice. The mice in the LPS‐challenged, vehicle‐treated group demonstrated a significant reduction in body temperature on day 2 of LPS challenge compared with that in unchallenged, untreated mice (*p* < 0.0005) and LPS‐challenged, SPA4 peptide‐treated mice (*p* < 0.05, Figure 9a). All surviving mice, in both groups, demonstrated only moderate changes in body temperature at later time points (Figure 9a).

The body weights of mice in the LPS‐challenged, SPA4 peptide‐treated and LPS‐challenged, vehicle‐treated groups remained unaltered on day 2 of LPS challenge compared with the unchallenged, untreated group. However, there was a statistically significant reduction in body weight within the first 3 days of LPS‐challenge in surviving mice (Figure 9b). These mice started to then gain body weight between days 8 and 10 of LPS‐challenge. The gain in body weights was more noticeable in surviving SPA4 peptide‐treated mice (*p* < 0.05 vs. unchallenged, untreated mice) than in vehicle‐treated mice (*p* < 0.0005 vs. unchallenged, untreated mice). Mice in the unchallenged, untreated group gained an average of 0.307 g body weight during the 10‐day study period. On day 10, the surviving mice in the LPS‐challenged, SPA4 peptide‐treated group lost an average of 1.381 g body weight, and mice in the LPS‐challenged, vehicle‐treated group lost 1.914 g body weight (Figure 9b).

**FIGURE 10:**
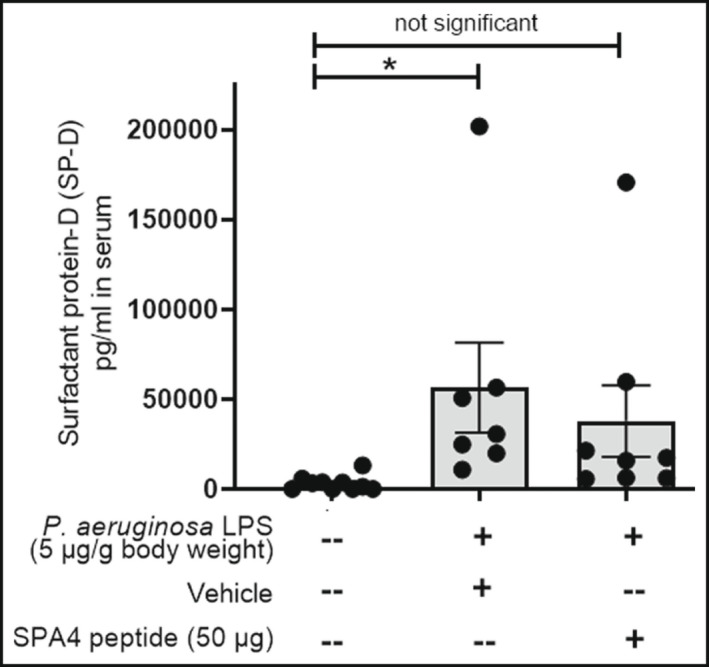
Levels of SP‐D (pg/ml) in serum specimens harvested from mice after the 10‐day survival study in mice challenged with *Pseudomonas aeruginosa* LPS and treated with vehicle or SPA4 peptide. The SP‐D levels were measured by ELISA in a blinded manner. Results (mean ± SEM) are from 7 to 10 mice per group included in two separate experiments. The data were analyzed using *t*‐test (*p < 0.05 vs. unchallenged, untreated mice).

The SP‐D levels were measured in serum as a marker of lung injury in mice surviving on day 10 of LPS‐challenge, and vehicle or SPA4 peptide treatment. The SP‐D levels were significantly increased in serum of surviving mice in the vehicle‐treated group as compared with that in unchallenged, untreated mice (*p* < 0.05). However, the serum SP‐D levels were not significantly different between the SPA4 peptide‐treated and unchallenged, untreated groups (Figure 10).

## 4 DISCUSSION

The SPA4 peptide exerts pro‐phagocytic and anti‐inflammatory activity in immune cells and in a mouse model of *P. aeruginosa*‐induced bacterial lung infection (Awasthi et al., 2019). We performed the present study to decipher the SPA4 peptide activity on barrier function of lung alveolar epithelial cells, and immunologic and physiologic outcomes in a mouse model against *P. aeruginosa*‐derived LPS stimuli. The SPA4 peptide reduces the inflammation induced by *E. coli*‐derived LPS, which expresses hexa‐acyl chains (Awasthi et al., 2020, 2021; Ramani et al., 2013). The extent of inflammation and damage could likely differ against different LPS species. Although *P. aeruginosa*‐derived LPS can contain both penta‐acylated and hexa‐acylated chains (Bedoux et al., 2004; Lam et al., 2011), *P. aeruginosa* 10 LPS used in this work is known to induce TLR4‐mediated inflammatory responses (Guillot et al., 2004; Lagoumintzis et al., 2008; Nocera et al., 2019; van der Plas et al., 2016).

The lung alveolar epithelial cells form an interface with the outside environment and serve as the first‐line barrier for inhaled pathogens and environmental antigens. Loss of barrier function and increased permeability are hallmarks of lung injury and inflammation, which contributes to pulmonary edema, hypoxemia, respiratory distress, and compromised lung function. We included human lung alveolar epithelial H441 cells, which formed a stable barrier under liquid–liquid interface. As expected, the lung alveolar epithelial barrier was disrupted by LPS challenge. The liquid–liquid interface mimics the biological scenario of fluid accumulation during lung inflammation (Leibrock et al., 2019; Lenz et al., 2013), and submerged epithelial blebs in alveolar edema fluid during injury and inflammation (Wu et al., 1995). Our results demonstrated that SPA4 peptide treatment significantly improved the LPS‐disrupted TEER and lung alveolar epithelial barrier function in a dose‐ and time‐dependent manner (100 μM SPA4 peptide; *p* < 0.05 and *p* = 0.0753 at 24 and 48 h, respectively, Figure 2). Since the H441 cells express an increased level of TLR4 even at basal level [Supplemental Figure in (Jeon et al., 2016), and (Rahman et al., 2021)], it is likely that an increased treatment dose (100 μM) was needed for restoration of the epithelial barrier.

Furthermore, our results revealed that the SPA4 peptide restores the LPS‐disrupted TEER by mainly affecting the subcellular distribution of tight junction proteins (Figure 3). Similarly, changes in localization pattern, but no effect on expression levels of occludin, have been reported earlier in human bronchial epithelial cells challenged with *E. coli* O111:B4‐derived LPS, pollutants, and cigarette smoke, through hyperphosphorylation of occludin (Cao et al., 2015; Ma et al., 2021). Similarly, tyrosine phosphorylation of ZO‐1, occludin, and E‐Cadherin results in their re‐distribution at a cellular level (Samak et al., 2014). The phosphorylation of tight junction proteins attenuates interaction with intracellular signaling molecules, leading to disruption of the barrier (Jain et al., 2011; Kale et al., 2003; Sheth et al., 2007). Among various intracellular signaling molecules, the protein kinase C (PKC) has been reported to affect the barrier function (Jain et al., 2011). The LPS‐induced TLR4‐signaling involves kinases such as IL‐1 receptor‐associated kinase (IRAK), PKC, mitogen‐activated protein kinase (MAPK), extracellular signal‐regulated kinase (ERK)1/2, p38‐MAPK, and c‐jun N‐terminal kinase (JNK) (Ciesielska et al., 2021; Loegering & Lennartz, 2011). In this regard, our results have demonstrated that the SPA4 peptide treatment reduces the phosphorylation of p54 SAPK/JNK, p38‐MAPK, and NF‐κB‐p65 in a mouse model of acute bacterial (*P. aeruginosa*) lung infection (Awasthi et al., 2019), and phosphorylation of NF‐κB‐p65 in a mouse model of *E. coli* LPS‐induced lung inflammation (Ramani et al., 2013).

The long‐term biological effects of SPA4 peptide remained unknown in models of lung inflammation. As expected, the LPS‐challenge induced lung edema (statistically significant at 24 and 48 h compared with unchallenged, untreated mice). The lung wet/dry weight ratios (indicator of lung edema) in SPA4 peptide‐treated mice were reduced and reached an equivalent level to those in unchallenged, untreated mice after 48 h (Figure 5). Similar results (reduced lung edema, but no statistical significance) were obtained at early time points after challenge with *E. coli* LPS and treatment with SPA4 peptide (Awasthi et al., 2020). However, a significant reduction in lung edema was noted within a short period of SPA4 peptide treatment in a mouse model of *P. aeruginosa*‐induced lung infection (Awasthi et al., 2019). An increased permeability during lung injury causes sustained intravascular leakage of alveolar SP‐D, resulting in an increased level of serum SP‐D (Gaunsbaek et al., 2013; Pan et al., 2002; Peukert et al., 2021). The SPA4 peptide treatment suppressed the LPS‐induced circulating levels of SP‐D (Figure 10).

An assessment of lung function parameters was included to assess the physiological effects of anti‐inflammatory activity of SPA4 peptide. The LPS‐induced changes in lung function parameters determined with the Flexivent instrument were consistent with published results (Mora et al., 2000; Rafikov et al., 2014; Verjans et al., 2018). The SPA4 peptide treatment improved the lung function parameters, specifically inspiratory capacity, compliance, curvature of the upper limb of pressure‐volume curves, and resistance, at 4 or 24 h of LPS challenge (Figure 6). In regards to general health parameters, the observed changes in body temperature and body weights in LPS‐challenged, vehicle‐treated mice corroborated with previously published results (Verjans et al., 2018). An improvement in some of the lung function and general health parameters correspondingly affected the 10‐day survival curves in LPS‐challenged and SPA4 peptide‐treated mice. The hazard ratio of 0.56 indicates that the SPA4 peptide‐treated mice have an approximately 44% lower risk of death (or 78% improvement in survival time) than do those mice treated with vehicle (Figure 8) [per (Barraclough et al., 2011)]. Together, the results presented here support the biological activity of SPA4 peptide against Gram‐negative bacteria and LPS stimuli (Awasthi et al., 2019, 2020). We speculate that repeated administration of SPA4 peptide could further improve its therapeutic efficacy in models of lung infection and inflammation. The repeated intratracheal administration of SPA4 peptide at least at 24 h intervals for 3 days is feasible because it does not induce toxicity or immunogenicity in healthy outbred mice (Awasthi et al., 2020). Our findings point towards performing detailed mechanistic and efficacy studies with SPA4 peptide for establishing its translational and therapeutic potential in lung infection and inflammation.

## AUTHORS’ CONTRIBUTIONS

Asif Alam Chowdhury performed UV–VIS spectrophotometry, membrane barrier function analysis, and immunocytochemistry to assess the localization of tight junction protein, and assisted with animal studies. Nachiket M. Godbole performed western immunoblotting and ELISA to analyze the expression of tight junction proteins and levels of SP‐D in serum. Neha Chataut analyzed the data on lung function parameters gathered by Flexivent and immunostaining in a blinded manner. Stanley Kosanke interpreted lung inflammation and damage parameters in H & E‐stained lung tissue sections. Karla Rodgers assisted and interpreted the CD analysis of SPA4 peptide. Shanjana Awasthi designed and coordinated the study; prepared SPA4 peptide for different analyses; performed intratracheal instillations, necropsy, and lung function analysis; and harvested BALFs and lung tissues from mice.

## FUNDING INFORMATION

Research reported here was supported in whole by the funding from National Heart, Lung, and Blood Institute of the National Institute of Health under award number R01 HL136325. The content is solely the responsibility of the authors and does not necessarily represent the official views of the National Institute of Health.

## CONFLICT OF INTEREST

The authors declare that they have no competing interests.

## ETHICS APPROVAL AND CONSENT TO PARTICIPATE

The animal studies were approved by the Institutional Animal Care and Use Committee (Protocol numbers: 17‐081‐CHR and 20‐054‐SCHIR) at the University of Oklahoma Health Sciences Center (OUHSC), Oklahoma City, OK, USA.
